# Supplementary material for: The Impact of Targeted Therapies on Red Blood Cell Aggregation in Patients with Chronic Lymphocytic Leukemia Evaluated Using Software Image Flow Analysis
Source: Micromachines (Basel). 2025 Jan 15;16(1):95. doi: 10.3390/mi16010095 (PMC11767778; doi:10.3390/mi16010095)
Supplement: Supplementary file 1 [file micromachines-16-00095-s001.zip › micromachines-3377027-supplementary.pdf]

The Impact of Targeted Therapies on Red Blood Cell Aggregation in Patients with Chronic Lymphocytic Leukemia Evaluated Using Software Image Flow Analysis

Anika Alexandrova-Watanabe [1,2,†], Emilia Abadjieva [1,2,†], Lidia Gartcheva [3], Ariana Langari [2,4], Miroslava Ivanova [4], Margarita Guenova [3], Tihomir Tiankov [1,2], Velichka Strijkova [5], Sashka Krumova [4] and Svetla Todinova [2,4,*]

[1] Institute of Mechanics, Bulgarian Academy of Sciences, "Acad. G. Bontchev" Str. 4, 1113 Sofia, Bulgaria; anikaalexandrova@abv.bg (A.A.-W.); abadjieva@gmail.com (E.A.); tiho_bg@abv.bg (T.T.)
[2] Center of Competence at Mechatronics and Clean Technologies—MIRACle, "Acad. G. Bontchev" Str. 4, 1113 Sofia, Bulgaria; arianalangari@abv.bg
[3] National Specialized Hospital for Active Treating of Hematological Diseases, Zdrave Str. 2, 1756 Sofia, Bulgaria; l.garcheva@hematology.bg (L.G.); m.genova@hematology.bg (M.G.)
[4] Institute of Biophysics and Biomedical Engineering, Bulgarian Academy of Sciences, "Acad. G. Bontchev" Str. 21, 1113 Sofia, Bulgaria; miroslava.ilieva.ivanova@gmail.com (M.I.); sashka.b.krumova@gmail.com (S.K.)
[5] Institute of Optical Materials and Technologies "Acad. Yordan Malinovski", Bulgarian Academy of
Sciences, "Acad. G. Bontchev" Str. 109, 1113 Sofia, Bulgaria; vily@iomt.bas.bg
* Correspondence: todinova@abv.bg
† These authors contributed equally to this work.

Supplementary Text 1. Microfluidic system description

The microfluidic system used in the experiments in the present study includes the following devices:

- BioFlux 200 electro-pneumatic flow control pump: used for controlling fluid flow within the microfluidic channels.
- Lumascope 620 inverted fluorescence microscope: provides high-resolution imaging for visualizing biological or chemical processes within the microfluidic system.
- BioFlux microfluidic plates: specialized plates designed for the flow and analysis of samples within a microfluidic environment.
- Computer configuration with specialized control and analysis software: enables precise control over the experimental conditions and facilitates the collection and analysis of data from the system.

The integral configuration is shown in Figure S1 and the top view of the BioFlux 24-well plates, along with details on channel positioning and channel dimensions, is shown in Figure S2.

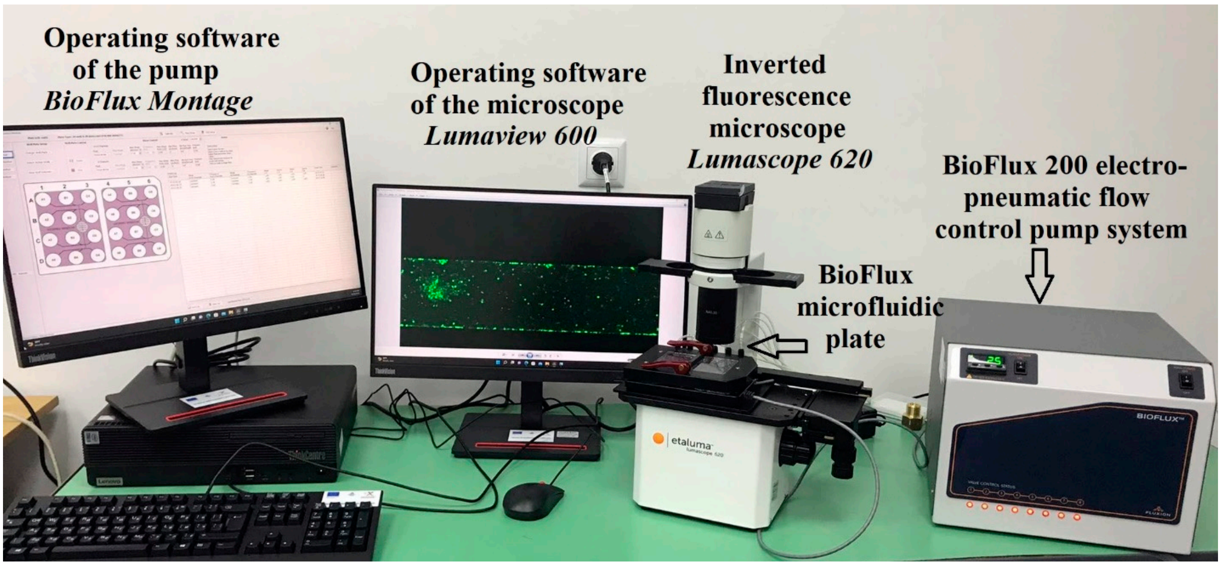

Figure S1. Photograph of the microfluidic system used in the experiments in the present study. The system includes the following devices: BioFlux 200 electro-pneumatic flow control pump, a Lumascope 620 inverted fluorescence microscope, BioFlux microfluidic plates, and a computer configuration with specialized control and analysis software.

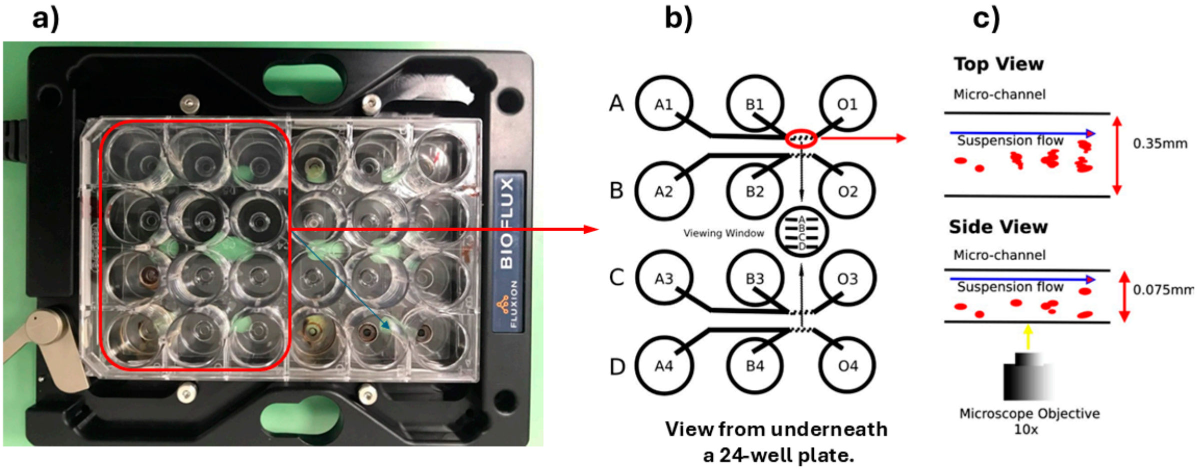

Figure S2. Top view of BioFlux 24-well plates 0–20 dyn/cm^2 including eight experimental channels (a); schematic imaging of half (12 wells, A1–A4, B1-B4, and O1–O4) of the BioFlux 24-well plates and the microfluidic channels (A,B,C, and D) located between pairs of experimental wells (b); and enlarged schematic image of a viewing part of a microfluidic channel (top and side view with the respective dimensions) with a schematic representation of the flow of red blood cells (c). Channel thickness—75 μm; channel width—350 μm.

Supplementary Text 2. Red Blood Cell Aggregation Characteristics

After applying the Software Image Flow analysis, the aggregates in the RBC suspensions of the control and CLL groups were classified into several distinct populations (P1–P5) of different sizes and were coded with their corresponding color (Figure S3).

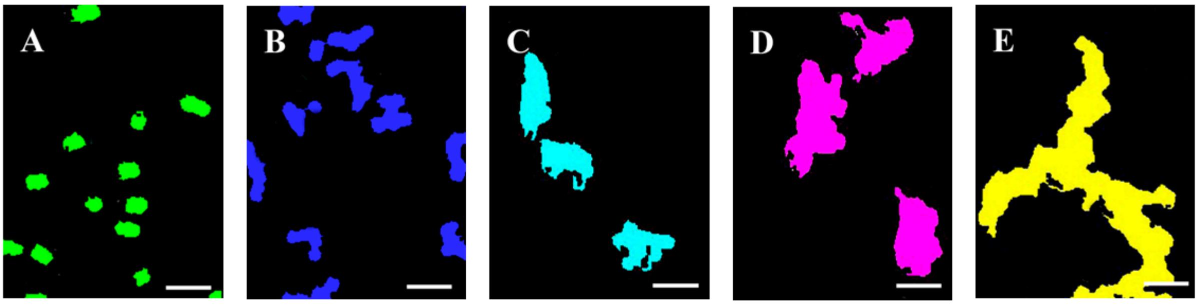

Figure S3. Representative images obtained after Software Image Flow analysis, corresponding to the selected individual aggregates from Fig. 2 and shown in increasing order of aggregation as follows: A) small linear rouleaux (100 – 330 μm^2); B) branched rouleaux (331 – 660 μm^2); C) medium 3D aggregates (661 – 1320 μm^2); D) large 3D aggregates (1321 – 2700 μm^2); and E) aggregate networks (>2700 μm^2). Each image is presented using the color code for RBC aggregate area range used in Fig. 4. Scale bar—25 μm.

Table S1 summarizes the red blood cell aggregation characteristics for untreated patients, patients receiving Ibrutinib, patients treated with Obinutuzumab/Venetoclax, and healthy controls. The parameters include the total number of aggregates, total area size, and area size percentage (ASP1 – ASP5) for different aggregate size ranges, calculated as the ration of the area of the aggregates from each population group (P1 – P5) relative to the total aggregate area. By calculating these percentages, one can assess the dominance of certain aggregate size ranges, which is important for understanding the behavior of RBCs under study.

Table S1. Red blood cell aggregation characteristics for one visual field of the microscope: total number of aggregates, total aggregate area size, and the area size percentage (ASP_1 – ASP_5) calculated for aggregates with sizes of <330 μm^2; 331 -666 μm^2; 661 – 1320 μm^2; 1321 – 2700 μm^2 and > 2700 μm^2 for the untreated patients, patients receiving Ibrutinib or Obinutuzumab/Venetoclax, and healthy controls, along with mean values and SD.

Parameters	Groups			
	**Healthy controls**	**CLL patients without treatment**	**CLL patients receiving Obinutuzumab/ Venetoclax**	**CLL patients receiving ibrutinib**
total aggregate area size (μm^2)	47387±2562	54996±2542	47508±2538	53042±2447
total number of aggregates	152.3±7.2	92.1±7.5*	99.5±8.3*	169.6±12.5
ASP_1 (%)	39.4 ± 6.7	15.8 ± 8.9*	23.9 ± 12.1*	40.2 ± 9.1
NAP_1 (%)	70.0 ± 4.1	49.4 ± 3.2	58.6 ± 7.1	68.3 ± 9.6
ASP_2 (%)	33.3 ± 3.2	17.3 ± 4.4*	23.3 ± 9.8*	30.5 ± 3.0
NAP_2 (%)	22.7 ± 5.2	22.3 ± 4.6	22.3 ± 6.7	20.4 ± 6.1
ASP_3 (%)	22.9 ± 10.8	20.8 ± 5.3	20.6 ± 6.6	23.1 ± 5.1
NAP_3 (%)	6.8 ± 2.3	13.6 ± 2.8	10.7 ± 4.0	9.1 ± 3.2
ASP_4 (%)	1.8 ± 0.4	24.3 ± 9.6*	16.3 ± 7.8*	6.2 ± 4.0
NAP_4 (%)	0.6 ± 0.2	7.3 ± 2.1	4.6 ± 1.9	2.2 ± 0.8
ASP_5 (%)	-	24.6 ± 10.8	15.3 ± 8.9	-
NAP_5 (%)	-	3.8 ± 1.2	2.1 ± 1.1	

* Indicates statistically significant difference ($p < 0.05$) in the values of ASP_i ($i = 1 \div 5$) and total NAP_i ($i = 1 \div 5$) for CLL patient groups compared with the control values.

Supplementary Text 3. *Relative contribution (in percentage) of the morphological types of RBCs derived from healthy controls, untreated CLL patients, CLL patients receiving Obinutuzumab/Venetoclax, and CLL patients receiving ibrutinib treatment.*

The morphology of freshly isolated RBCs from healthy controls, untreated CLL patients, and CLL patients receiving Obinutuzumab/Venetoclax or ibrutinib treatment was characterized via optical microscopy. The distribution of three typical morphological types, designated as biconcave, echinocyte, and spherocyte, in fresh red blood cells is presented in Figure S4, and their relative contribution, expressed using smears from the different types of samples, is presented in Figure S5.

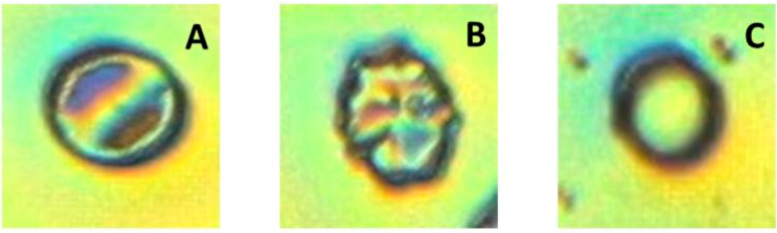

Figure S4. Typical optical images of morphological classes of red blood cells: (A) biconcave disk; (B) echinocyte; (C) spherocyte.

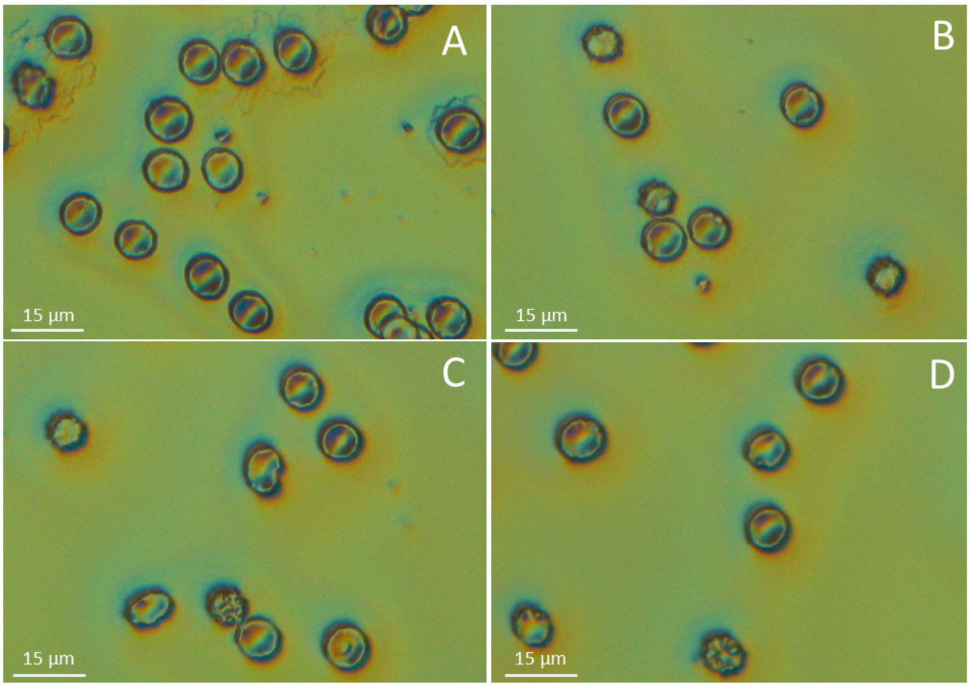

Figure S5. Representative images obtained using optical microscopy of smears of freshly isolated RBCs from healthy controls (A), untreated CLL patients (B), CLL patients receiving Obinutuzumab/Venetoclax (C), and CLL patients receiving Ibrutinib (D). Scale bar—15 µm.
